# Follicular T cells in the ovarian cancer immune microenvironment: biological insights and translational implications

**DOI:** 10.3389/fimmu.2026.1849305

**Published:** 2026-06-02

**Authors:** Yidan Xu, Li Li

**Affiliations:** 1 The Third Clinical Medical College of Xinjiang Medical University (Affiliated Tumor Hospital), Urumqi, China; 2 Department of Gynecology, Affiliated Tumor Hospital of the Third Clinical Medical College, Xinjiang Medical University, Urumqi, China

**Keywords:** follicular T cells, immunotherapy, ovarian cancer, tertiary lymphoid structures, tumor immune microenvironment

## Abstract

**Background:**

Ovarian cancer, particularly high-grade serous ovarian cancer (HGSOC), exhibits a generally poor response to immune checkpoint inhibitors, and its underlying mechanisms remain incompletely defined. This observation suggests that an immunological framework focused predominantly on CD8^+^ cytotoxic T cells is insufficient to fully explain immune resistance in ovarian cancer. Increasing evidence highlights the importance of tertiary lymphoid structures (TLS) and follicular immune responses in coordinating antitumor immunity and shaping therapeutic outcomes.

**Main body:**

Follicular T cell subsets—including follicular helper T cells (Tfh), follicular regulatory T cells (Tfr), and CXCR5^+^ follicular-like cytotoxic CD8^+^ T cells (Tfc)—form a dynamic immunoregulatory axis that governs B-cell activation, germinal center–like structure formation, antigen presentation, and local immune microenvironment remodeling. In this review, we systematically summarize the differentiation programs and regulatory mechanisms of follicular T cells and their compartment-specific distribution and functional remodeling in ovarian cancer. We integrate current evidence regarding their interactions with TLS maturation, B-cell function, and effector CD8^+^ T cell responses. Attention is given to the emerging concept that dysregulation of the follicular T cell axis—especially an elevated Tfr/Tfh ratio and defective functional maturation of TLS—may contribute to immune suppression and resistance to immunotherapy in ovarian cancer. From a translational perspective, we discuss therapeutic strategies aimed at reprogramming follicular T cell responses, including immune checkpoint modulation, chemokine axis targeting, metabolic interventions, and engineered cell-based therapies. We also highlight follicular immune features as potential biomarkers for immunotherapy response prediction and patient stratification.

**Conclusions:**

Collectively, this review proposes a follicular T cell–centered immunological framework that extends beyond conventional CD8^+^ T cell–centric models and identifies promising therapeutic avenues to overcome immunotherapy resistance in ovarian cancer.

## 1 Introduction

Ovarian cancer, particularly high-grade serous ovarian cancer (HGSOC), remains one of the most lethal malignancies of the female reproductive system (1). Despite high initial response rates achieved with standard first-line treatment consisting of cytoreductive surgery followed by platinum-based chemotherapy, most patients ultimately experience disease recurrence and progression to chemotherapy-resistant states, resulting in limited long-term survival improvement (2, 3). This persistent clinical challenge has gradually shifted research focus from tumor cell–intrinsic targets toward a more comprehensive understanding of the tumor immune microenvironment (TME) and the mechanisms underlying immune dysregulation in ovarian cancer. The immune landscape of HGSOC is characterized by pronounced functional paradoxes. On the one hand, infiltration of CD8^+^ T cells within tumor tissues are consistently associated with improved clinical outcomes, indicating the presence of latent antitumor immune potential (4, 5). On the other hand, immunosuppressive components—including regulatory T cells (Tregs) and tumor-associated macrophages (TAMs)—are highly enriched and cooperatively constrain the durability and effectiveness of effector immune responses (6). This immunosuppressive context is widely regarded as a major contributor to the limited efficacy of immune checkpoint inhibitors (ICIs) in ovarian cancer, suggesting that therapeutic strategies aimed solely at releasing effector T-cell inhibition may be insufficient to fully restore antitumor immunity (6–8).

Notably, tertiary lymphoid structures (TLS), which are frequently observed in ovarian cancer tissues, provide an important spatial framework for local adaptive immune responses (9, 10). TLS are ectopic lymphoid aggregates that develop postnatally in non-lymphoid tissues, and their functional maturation follows a conserved three-stage trajectory: uncompartmentalized precursors, primary TLS with distinct T/B zones but no FDC network, and mature GC-type TLS with CD21^+^CD23^+^ FDC networks and active GC reactions (11, 12). These observations imply that, beyond classical cytotoxic immunity, humoral immune responses and their spatial organization may play underappreciated roles in shaping antitumor immunity in ovarian cancer. Importantly, these findings suggest that the immunological significance of TLS may depend less on their mere presence than on their internal cellular composition and functional state—particularly whether a competent follicular immune regulatory axis is effectively established.

Follicular T cells are a specialized group of T lymphocytes characterized by expression of the C-X-C chemokine receptor type 5 (CXCR5), which enables their migration into B-cell follicles and germinal centers (GCs) (13). This population primarily includes follicular helper T cells (Tfh), follicular regulatory T cells (Tfr), and the more recently described CXCR5^+^ follicular-like cytotoxic CD8^+^ T cells (Tfc) (14–17). In the tumor context, however, follicular T cells exhibit pronounced functional duality. Tfh cells may promote antitumor immunity by supporting humoral responses and enhancing CD8^+^ T-cell effector function, whereas expansion of Tfr cells or functional impairment of Tfh cells may be exploited by tumors to reinforce local immunosuppression (18–20). In contrast, the role of Tfc cells in solid tumors remains incompletely defined. In ovarian cancer, current understanding of Tfc function is largely extrapolated from studies in other tumor types (21, 22), with direct experimental evidence still limited.

The unique biological features of ovarian cancer—including its peritoneal dissemination pattern, ascites enriched in immune cells and soluble mediators, and relatively frequent yet functionally heterogeneous TLS formation—provide a distinct context for follicular T-cell differentiation, localization, and functional remodeling (23, 24). Nevertheless, existing studies have predominantly focused on Tfh cells (25–27), while systematic characterization of Tfr and Tfc subsets remains insufficient. Consequently, whether follicular T cells primarily facilitate antitumor immunity or contribute to immune evasion in ovarian cancer remains unresolved.

Against this background, the present review integrates current knowledge of follicular T-cell biology with a critical appraisal of evidence specific to ovarian cancer. By synthesizing data on the distribution and functional states of follicular T cells across tumor tissues, ascites, and peripheral blood, we delineate their networked interactions with B cells, antigen-presenting cells (APCs), and effector T cells. Finally, we discuss potential therapeutic strategies targeting the follicular T-cell axis and address the translational challenges associated with harnessing follicular immunity to improve immunotherapeutic outcomes in ovarian cancer.

## 2 Follicular T cell subsets: biological foundations

**Figure 1:**
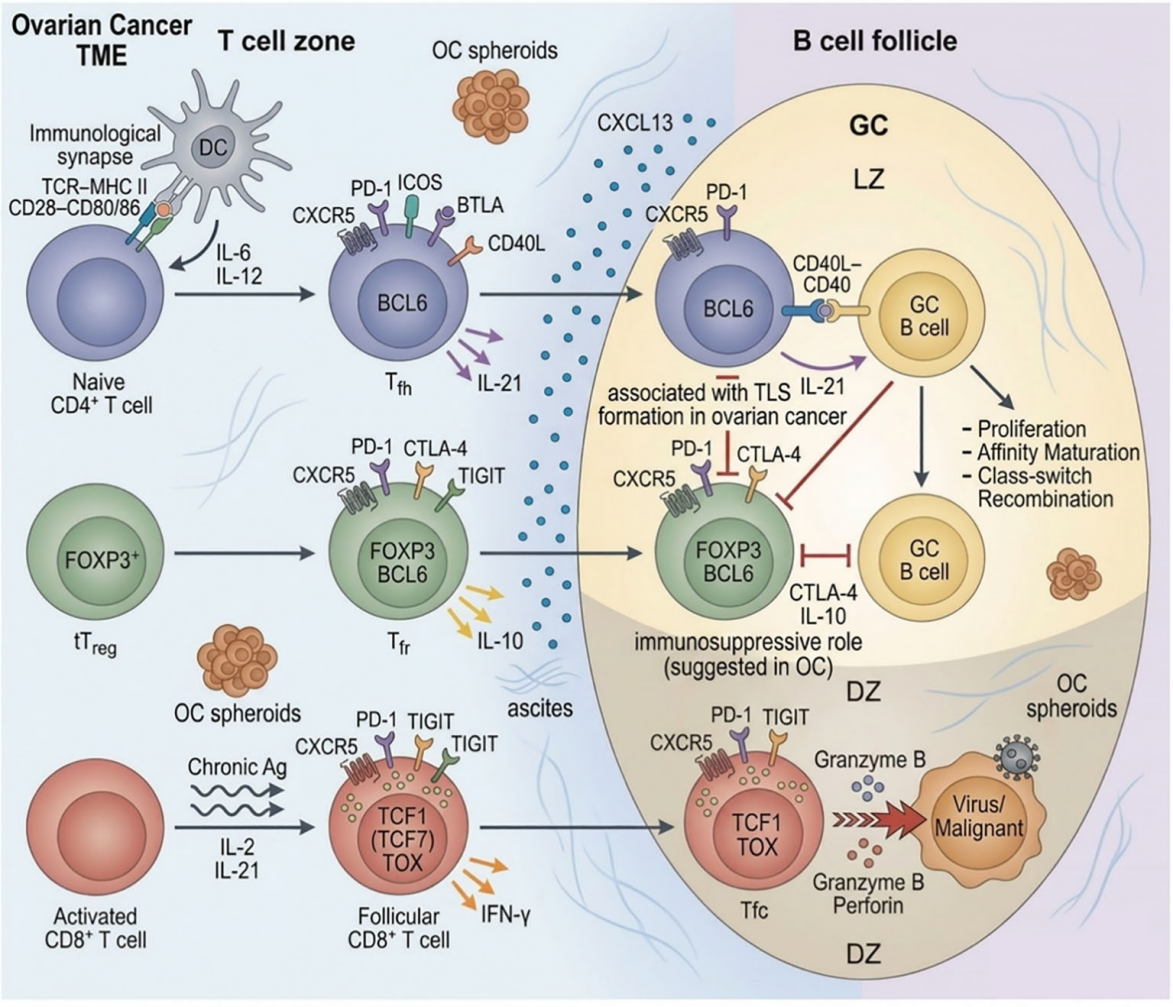
Follicular T cell subsets in the germinal center. Naïve CD4^+^ T cells differentiate into T follicular helper (Tfh) cells upon dendritic cell priming and B cell interaction, driven by ICOS signaling, IL-6/IL-21, and the transcription factor BCL6. Thymus-derived FOXP3^+^ regulatory T cells give rise to follicular regulatory T (Tfr) cells that co-express FOXP3 and BCL6 and suppress excessive germinal center reactions. Under chronic antigen stimulation, activated CD8^+^ T cells differentiate into CXCR5^+^ follicular cytotoxic T (Tfc) cells, which infiltrate B cell follicles and eliminate infected or malignant B cells. Together, these follicular T cell subsets coordinate humoral immunity by providing help, enforcing regulation, and maintaining immune surveillance within the germinal center.

Under physiological conditions, these subsets cooperatively regulate B-cell differentiation, GC reactions, and antibody maturation, thereby maintaining humoral immune homeostasis (28–30). Through responsiveness to C-X-C motif chemokine ligand 13 (CXCL13) produced by follicular dendritic cells (FDCs) and B cells, CXCR5^+^ T cells overcome the conventional T–B zone boundary and access follicular microenvironments (31, 32). Based on developmental origin, functional properties, and regulatory programs, follicular T cells are broadly classified into three major subsets: Tfh, Tfr, and Tfc. Although these subsets share overlapping spatial localization within follicles or TLS, they exert complementary yet distinct roles in immune activation, regulation, and cytotoxic surveillance (**Figure 1**).

### 2.1 Tfh cells

Tfh cells originate from naïve CD4^+^ T cells and undergo a multistep differentiation process tightly regulated by APCs and cytokine cues (33, 34). Integration of these signals induces upregulation of the transcriptional repressor B-cell lymphoma 6 protein (BCL6), the primary master regulator of the Tfh lineage in most physiological contexts. BCL6 suppresses alternative CD4^+^ T-cell differentiation programs (Th1, Th2, Th17) while promoting expression of follicular homing molecules (CXCR5, PD-1) (35).

Phenotypically, mature Tfh cells are characterized by high expression of CXCR5, which mediates follicular localization, along with elevated levels of activation and regulatory markers such as PD-1, ICOS, and BTLA, coupled with low expression of CCR7 (36, 37). Functionally, Tfh cells are indispensable drivers of GC reactions. Through secretion of IL-21 (and, in specific contexts, IL-4) and engagement of CD40L, Tfh cells directly support B-cell survival, clonal expansion, somatic hypermutation, and immunoglobulin class-switch recombination, ultimately shaping the generation of high-affinity plasma cells and memory B cells (38, 39). Dysregulation of Tfh cell number or function is therefore closely linked to the pathogenesis of autoimmune and antibody-mediated diseases.

### 2.2 Tfr cells

Tfr cells are currently thought to arise primarily from thymus-derived forkhead box P3 (FOXP3^+^) regulatory T-cell precursors. In contrast to Tfh cells, however, FOXP3 remains the dominant lineage-defining transcription factor in Tfr cells and continuously enforces immunosuppressive programs (20). Accordingly, Tfr cells exhibit a hybrid molecular phenotype, combining follicular homing markers such as CXCR5 and PD-1 with classical regulatory features including FOXP3, CTLA-4, and IL-10 expression. Functionally, Tfr cells restrain excessive Tfh activity, limit B-cell expansion, and suppress antibody production, thereby preventing uncontrolled GC responses. Through these mechanisms, Tfr cells play a critical role in maintaining humoral immune tolerance and protecting against the development of autoreactive antibody responses (40, 41).

### 2.3 Tfc cells

Tfc constitute a recently defined subset of CXCR5-expressing effector CD8^+^ T cells. Rather than representing a lineage distinct from conventional CD8^+^ T-cell differentiation pathways (21), Tfc cells arise from peripherally activated CD8^+^ T cells that acquire CXCR5 expression under conditions of chronic antigen stimulation, such as persistent viral infection or sustained tumor antigen exposure. This process typically occurs within secondary lymphoid organs and confers follicular homing capacity (42). Phenotypically, Tfc cells are characterized by CXCR5 expression and retention of cytotoxic machinery, including granzyme B and perforin, while simultaneously displaying high levels of activation- and exhaustion-associated markers such as PD-1, TIGIT, and TOX. This unique phenotype enables Tfc cells to infiltrate B-cell follicles and GCs—anatomic niches that are relatively inaccessible to conventional effector CD8^+^ T cells—and to exert immune surveillance within these so-called immune-privileged regions (43).

## 3 Follicular T cells in ovarian cancer

Under physiological conditions, follicular T cells—including Tfh, Tfr, and the more recently described Tfc subset—primarily participate in GC reactions to finely regulate B-cell differentiation and antibody maturation. In ovarian cancer, a disease characterized by marked immune heterogeneity, ascites formation, and chronic antigen stimulation, this cellular compartment undergoes substantial remodeling in terms of abundance, spatial distribution, and functional state. Accumulating evidence indicates that the role of follicular T cells in ovarian cancer is highly context dependent and strongly influenced by their anatomical localization, including peripheral blood, tumor tissue, and ascitic fluid.

### 3.1 Distribution across immune compartments

Studies consistently demonstrate that follicular T cells are detectable across multiple immune compartments in ovarian cancer patients, yet their distribution patterns and functional properties differ markedly. In primary tumor tissues, Tfh cells tend to co-occur with exhausted CD8^+^ T cells and CD4^+^ Tregs, while showing negative correlations with naïve CD4^+^/CD8^+^ T cells and effector CD8^+^ T-cell subsets (44). These associations suggest that follicular T cells are preferentially enriched in immunologically experienced and chronically stimulated tumor niches. In peripheral blood, ovarian cancer patients exhibit significantly increased proportions of CD4^+^CD25^+^CD127^-^CXCR5^+^ T cells and CD4^+^CD25^+^CD127^-^CXCR5^+^FOXP3^+^ cells compared with healthy controls. Functional analyses further reveal that circulating Tfr cells from ovarian cancer patients express elevated levels of TGFB1 and IL10 *in vitro*, indicating a systemic bias toward immunosuppressive regulation even outside the TME (45). These findings suggest that ovarian cancer–associated immune dysregulation extends beyond the tumor site and is reflected in peripheral immune compartments.

Within the TME, follicular T cells undergo further functional adaptation. The frequency of PD-1^+^ Tfh cells, as well as TIM-3^+^PD-1^+^ Tfh subsets, is significantly higher in tumor-infiltrating lymphocytes than in peripheral blood, consistent with sustained antigen exposure and chronic activation (46). This phenotype is often accompanied by functional constraints, suggesting partial exhaustion or altered helper capacity. Tfr cells are also readily detectable in tumor tissues and ascites, where they are enriched relative to peripheral blood and maintain expression of immunosuppressive cytokines such as IL-10 and TGF-β (45). These cytokines have been shown to inhibit CD8^+^ T-cell activation and cytotoxic function, thereby contributing to the establishment and maintenance of an immunosuppressive TME.

Collectively, these observations indicate that follicular T cells in ovarian cancer are not merely altered in number but are profoundly reshaped by compartment-specific cues. In particular, the ascitic milieu—enriched in immunosuppressive cytokines, extracellular vesicles, and persistent antigenic stimulation—may represent a permissive yet functionally restrictive niche for follicular T-cell differentiation and effector output.

### 3.2 Association with TLS

The functional impact of follicular T cells is tightly linked to their spatial organization, particularly within TLS. In tumors with high mutational burden, enhanced Tfh differentiation has been associated with TLS expansion and increased B-cell recruitment (47, 48). In HGSOC, however, the composition and maturation status of TLS exhibit substantial heterogeneity. Compared with non–small cell lung cancer (where TLS maturation is generally more robust), HGSOC tumors display significantly lower densities of CXCR5^+^PD-1^+^FOXP3^-^ Tfh cells, accompanied by reduced proportions of CD4^+^ T cells, CD8^+^ T cells, and Tfh cells within mature TLS. Moreover, the density of CD21^+^CD23^+^ FDCs—critical organizers of GC reactions—is markedly diminished (10). These cellular defects directly underlie the pre-GC arrest of HGSOC-associated TLS. Only mature GC-type TLS, defined by CD21^+^ FDC networks and BCL6^+^ GC B cells, are independently associated with prolonged survival, while an elevated intratumoral Tfr/Tfh ratio predicts suppressed GC formation and poor prognosis. These observations underscore that TLS functional competence is determined by both Tfh abundance and the Tfr/Tfh balance within the follicular niche (49, 50). Importantly, TLS detected in ovarian cancer often display incomplete maturation, characterized by reduced FDC networks and diminished Tfh–B cell interactions compared with TLS in lung or colorectal cancer. This qualitative difference may explain why the mere presence of TLS does not uniformly predict favorable outcomes, underscoring the importance of functional follicular immune organization rather than structural presence alone.

### 3.3 Clinical relevance and prognostic implications

High-resolution immune profiling approaches have provided further support for the functional relevance of the follicular immune axis in ovarian cancer. Paired single-cell RNA sequencing and TCR sequencing analyses reveal that Tfh-like cells—particularly TCF7^+^ subsets—are preferentially activated and reprogrammed during ex vivo expansion, exhibiting self-renewal capacity and sustained tumor reactivity both *in vitro* and *in vivo* (51). Mechanistically, Tfh cells constitute a major source of CXCL13, especially within TLS, where they spatially colocalize with CD20^+^ B cells (52). CXCL13 not only promotes B-cell recruitment but also increases the frequency of CXCR5^+^CD8^+^ T cells and enhances their expression of effector molecules such as granzyme B and interferon-gamma (IFN-γ). Spatial transcriptomic analyses of HGSOC tumors demonstrate that CXCR5^+^CD8^+^ T cells preferentially localize in proximity to CXCL13^+^ cells. In patients with detectable TLS, colocalization of CXCL13 and CXCR5^+^CD8^+^ T cells are significantly associated with improved survival, whereas this relationship is absent in TLS-negative tumors (53). Consistently, The CXCL13^high^/CXCR5^high^/CD8^high^ immune signature has been reported to be associated with favorable clinical outcomes in ovarian cancer, although these findings are primarily derived from retrospective and bioinformatic analyses and require further prospective validation. In lung, colorectal and pancreatic cancers, increased infiltration of CXCR5^+^CD8^+^ T cells (Tfc) correlates with improved patient survival (53–56). However, these findings have not been replicated in ovarian cancer, and whether Tfc cells retain cytotoxic capacity within ovarian cancer follicular niches or instead adopt exhausted phenotypes remains entirely unknown.

Collectively, current evidence indicates that the abundance, subset composition, and spatial association of follicular T cells with TLS are closely linked to clinical outcomes and immunotherapy responsiveness in ovarian cancer. These findings suggest that conventional metrics of immune cell infiltration alone may be insufficient to capture the functional immune status of tumors. Incorporation of follicular T-cell–related features—particularly the Tfh/Tfr balance and TLS maturation status—into immune classification frameworks may enable more precise identification of patient subsets with potential benefit from immunotherapeutic interventions (57). From a translational perspective, this follicular immune signature holds promise as a candidate biomarker for predicting ICI efficacy and guiding patient stratification in ovarian cancer. Notably, direct evidence for Tfc function in ovarian cancer remains limited, with most insights extrapolated from other solid tumors. Whether ovarian cancer-associated Tfc cells retain cytotoxic capacity within follicular niches or adopt exhausted phenotypes requires urgent experimental validation.

To clarify the current evidence, we summarized key studies on follicular T cells in ovarian cancer (**Table 1**), which highlights that clinical associations are increasingly reported but mechanistic validation remains scarce.

**Table 1:** Mechanistic roles of follicular T cells sebset in ovarian cancer.

Year	Cohort/model	Cell subset	Key findings	Mechanistic insight	References
2018	OC patients and healthy controls (Peripheral blood)	Tfh	OC PD-1^+^ Tfh ↑; TIM-3 exclusively on PD-1^+^ Tfh; TIM-3^+^PD-1^+^ Tfh function impaired.	TIM-3 blocks Tfh-B crosstalk via IL-21 inhibition, mediating immune escape	(46)
2019	OC patient (tumor, ascites, peripheral blood)	Tfr	Tfr enriched in OC tumor/ascites; Tfr highly secrete IL-10; Tfr suppress CD8^+^ T cell function; High Tfr → poor prognosis.	Tfr induces CD8^+^ T dysfunction via IL-10 pathway, mediating OC immune suppression	(45)
2020	TCGA EOC	Tfh	High Tfh → low risk; Favorable OS/PFS; Higher immunophenoscore.	Tfh marks active anti-tumor immunity; TME heterogeneity drives EOC prognosis.	(49)
2021	HGSOC patient, murine model	Tfh, Tfc	Tfh is major CXCL13 source; CXCL13 recruits Tfh/Tfc.	CXCL13-CXCR5 axis recruits Tfh/Tfc to shape immunoactive TME, sensitizing HGSOC to PD-1 blockade	(53)
2021	TCGA OC	Tfh	Tfh infiltration differs between high/low-risk groups; Tfh correlates with risk score.	Tfh enrichment indicates favorable immune microenvironment in OC	(111)
2022	TCGA HGSOC (LTS vs STS)	Tfh	LTS has significantly higher Tfh infiltration than STS	Increased Tfh infiltration associates with favorable long-term survival in HGSOC	(112)
2024	HGSOC patient	Tfh	HGSOC has low Tfh density; Tfh density correlates with TLS maturation.	Low Tfh impairs TLS function and CD8^+^ T cell effector maintenance in HGSOC	(10)
2024	TCGA OC	Tfh	High NETs risk score → decreased Tfh infiltration	NETs signature correlates with suppressed Tfh-mediated anti-tumor immunity	(113)
2025	OC scRNA-seq + bulk transcriptomics	Tfh	High crotonylation risk score → decreased Tfh infiltration	Crotonylation dysregulation suppresses Tfh-mediated anti-tumor immunity	(114)
2025	HGSOC patient, murine model	Tfh	NACT recruits Tfh; T fh required for mature TLS formation; TLS correlates with ICI-responsive TCF1^+^CD8^+^ T cells.	NACT drives Tfh infiltration to promote TLS maturation, sensitizing HGSOC to PD-1 blockade	(103)
2025	*In vitro* Tfr cell	Tfr	VISTA^+^ Tfr suppresses CD8^+^ T function; Inhibits B cell IgE secretion; Shifts Th balance to Th2.	VISTA enhances Tfr immunosuppressive activity, mediating OC immune escape	(79)
2025	EOC patient TIL	Tfh-like	TCF7^+^ Tfh-like preferentially expanded; Robust tumor reactivity; Enriched in CCR7^+^CD200^+^ T cell subset.	TCF7^+^ Tfh-like contributes to effective TIL therapy in OC	(51)
2026	TCGA OC	Tfh	Tfh infiltration differs OC vs normal; Prognostic genes negatively correlate with Tfh.	Nectin/Necl dysregulation associates with suppressed Tfh-mediated anti-tumor immunity	(115)

OC, ovarian cancer; HGSOC, high-grade serous ovarian cancer; Tfh, follicular helper T cells; Tfr, follicular regulatory T cells; Tfc, follicular cytotoxic T cells; TLS, tertiary lymphoid structures; TME, tumor immune microenvironment; ICI, immune checkpoint inhibitor; NACT, neoadjuvant chemotherapy.

## 4 Network interactions shaping follicular immunity

The immunological functions of follicular T cells within the TME are not determined by isolated, cell-intrinsic programs. Instead, they emerge from dynamic interactions within a multicellular immune network comprising B cells, APCs, effector T cells, and regulatory immune populations. Increasing evidence indicates that the integrity, directionality, and spatial organization of this network critically shape humoral immune responses, govern the formation and maintenance of TLS, and ultimately influence the strength and durability of cytotoxic antitumor immunity (58). Although direct mechanistic evidence in ovarian cancer remains limited, insights derived from other solid tumors and chronic inflammatory or infectious models provide a robust conceptual framework for understanding how follicular T-cell programs may operate in this disease context.

### 4.1 Interactions with B cells

Interactions between follicular T cells and B cells constitute the core regulatory axis of humoral immunity and represent a principal driver of TLS formation and functional maturation (59, 60). Within tumors, this axis does not merely determine whether B cells are activated, but also dictates the qualitative features of antibody responses, the efficiency of GC reactions, and the spatial organization of immune cell aggregates.

#### 4.1.1 Tfh–B cell interactions

**Figure 2:**
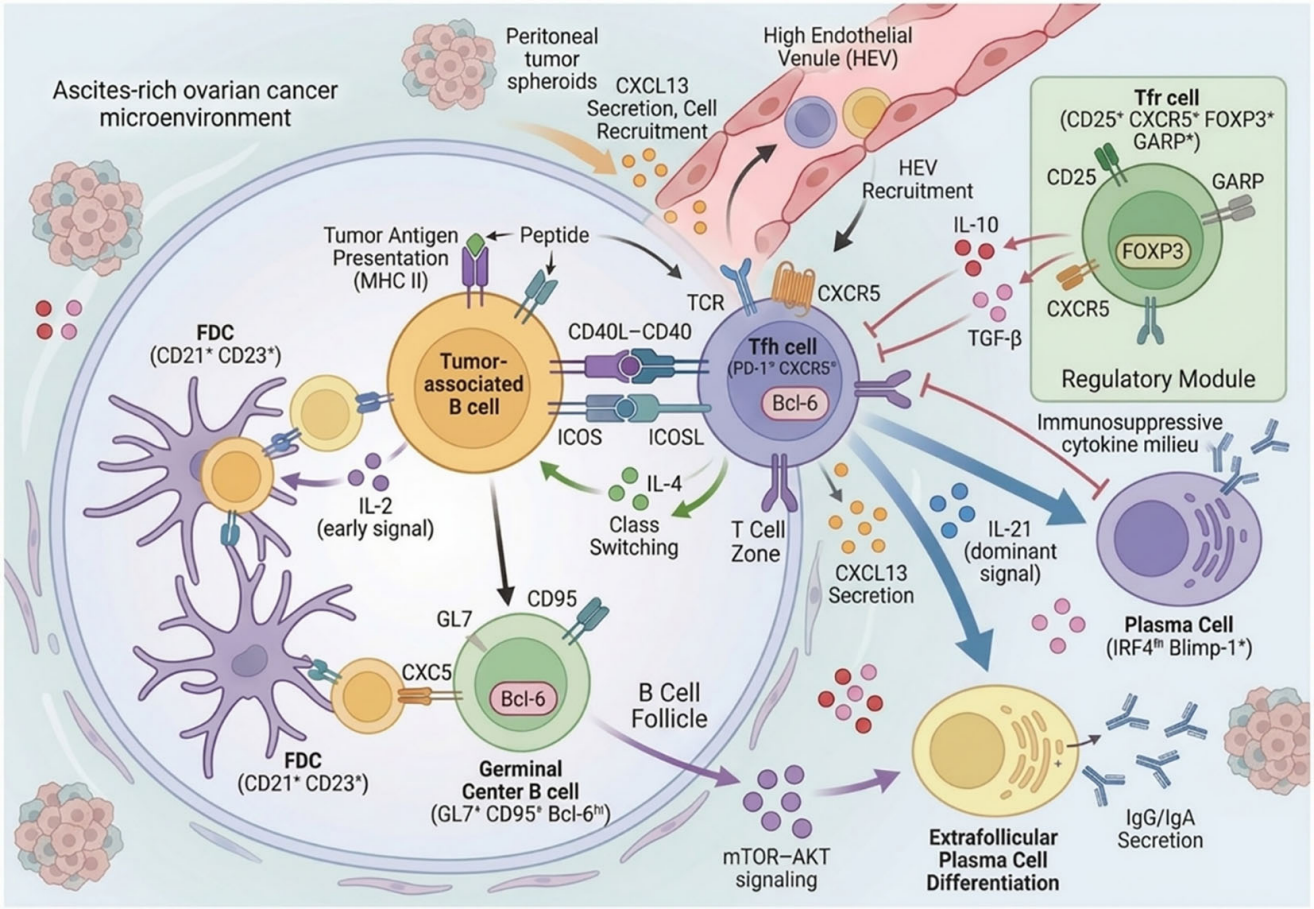
T follicular helper cell–B cell interactions within the ovarian cancer microenvironment. Within the ovarian cancer tumor microenvironment, particularly in the context of ascites, antigen-experienced B cells recognize tumor-associated antigens and present peptide–MHC class II complexes to CXCR5^+^ PD-1^+^ BCL6^+^ T follicular helper (Tfh) cells through CD40–CD40L and ICOS–ICOSL signaling. Activated Tfh cells secrete IL-21 as a dominant effector cytokine, promoting germinal center B cell differentiation, antibody class switching, and plasma cell generation, while also supporting CD8^+^ T cell function. Tfh-derived CXCL13 contributes to the recruitment of CXCR5^+^ immune populations and the spatial organization of tertiary lymphoid structures (TLS). A defining feature of ovarian cancer is the presence of an ascites-rich microenvironment and peritoneal dissemination, often characterized by floating tumor spheroids. Within this milieu, immunosuppressive cytokines such as IL-10 and TGF-β are enriched and can originate not only from T follicular regulatory (Tfr) cells but also from the broader tumor microenvironment. Tfr cells further constrain Tfh-mediated help via IL-10– and TGF-β–dependent mechanisms. The interplay between Tfh-driven immune activation and Tfr-mediated suppression, under these ovarian cancer–specific conditions, critically shapes TLS function and local antitumor immunity.

Tfh cells orchestrate B-cell activation, differentiation, and fate commitment through a coordinated network of co-stimulatory signals—most prominently CD40L–CD40 and ICOS–ICOSL—together with cytokines such as IL-21, IL-4, and IL-2. This signaling circuitry represents the central engine of humoral immune responses and underlies GC dynamics and TLS development (**Figure 2**). In ovarian cancer, while direct mechanistic studies remain relatively scarce, available evidence suggests that the Tfh–B cell axis is not absent but functionally constrained or reprogrammed, with its immunological output being highly contingent on the local immune milieu and the activation state of Tfh cells.

Notably, Tfh-mediated B-cell help is functionally heterogeneous. PD-1^+^ Tfh cells isolated from ovarian cancer patients retain the capacity to induce IgM, IgG, and IgA production when co-cultured with autologous naïve B cells, indicating preservation of baseline helper activity. In contrast, TIM-3^+^PD-1^+^ Tfh cells display a markedly reduced ability to support immunoglobulin secretion (46). These observations suggest that, in ovarian cancer, the functional quality of Tfh cells—rather than their numerical abundance—may be the dominant determinant of humoral immune output and its potential contribution to antitumor immunity. This distinction provides a mechanistic explanation for the frequent coexistence of detectable antibody responses with inadequate immune control in a subset of patients.

Effective Tfh–B cell cooperation relies on antigen-specific, bidirectional signaling. B cells capture tumor-associated antigens via the B-cell receptor, process them, and present peptide–MHC class II complexes to CD4^+^ T cells, thereby promoting Tfh differentiation. Mature Tfh cells, in turn, reinforce B-cell commitment to the GC lineage through CD40–CD40L, ICOS–ICOSL, and IL-21 signaling, sustaining the GC reaction (61–63). These findings highlight a reciprocal dependency that may be particularly relevant in ovarian cancer, where antigen availability and B-cell antigen-presenting capacity are frequently compromised. Beyond IL-21–mediated support of GC reactions, follicular T cells also shape B-cell fate decisions through IL-2 (38). This temporal cytokine regulation suggests that Tfh cells actively balance the speed versus fidelity of humoral immune responses. In ovarian cancer, such modulation may influence whether antibody responses remain short-lived and inflammatory or evolve toward high-affinity repertoires with greater antitumor potential.

In multiple solid tumor models, Tfh cells appear to be central orchestrators of TLS maturation: they first secrete CXCL13 to establish chemokine gradients that recruit CXCR5^+^ B cells, FDC precursors and Tfc cells to tumor sites, then localize to the T-B border to deliver CD40L and ICOS co-stimulatory signals that drive T/B zone compartmentalization, and finally produce IL-21 and IL-4 to induce BCL6 expression in B cells and support FDC network maturation, culminating in functional GC formation (64–66). In colorectal and ovarian cancer models, Tfh deficiency results in poorly matured TLS and attenuated antitumor immunity, whereas supplementation with antigen-specific CD4^+^ T cells restore TLS formation and tumor control (15, 47). These findings emphasize that the Tfh–B cell axis functions most effectively when embedded within organized immune structures rather than operating in isolation. Inflammatory cues further modulate the direction of Tfh–B cell cooperation. In antiviral immunity, blockade of IFN-γ signaling increases Tfh abundance, enhances GC B-cell formation, and augments antibody production (67), indicating that cytokine environments indirectly shape B-cell fate by regulating Tfh differentiation. Taken together, these mechanisms suggest that, in ovarian cancer, the clinical relevance of the Tfh–B cell axis likely depends on its integration into structurally intact, functionally mature TLS with limited immunosuppressive pressure, rather than its mere presence within inflamed or ascitic environments.

#### 4.1.2 Tfr–B cell interactions

Tfr cells are the primary negative regulators of TLS maturation, acting through CTLA-4-mediated competition for co-stimulatory signals, TGF-β/IL-10-dependent disruption of Tfh-B cell cognate interactions at the T-B border, and inhibition of FDC precursor differentiation (68–71). Within tumors, this regulatory program may be co-opted and amplified. Notably, the local Tfh/Tfr balance appears to be more informative than the absolute abundance of either subset alone in reflecting immune activity (72). In mouse models lacking Tfr cells or neuropilin expression in FOXP3^+^ cells, early accumulation of aberrant plasma cells occurs within GCs, accompanied by the generation of autoantibodies. Neuropilin suppresses BLIMP-1 while promoting BCL6 expression, thereby limiting plasma cell differentiation; restoration of neuropilin signaling reverses these abnormalities (18). In ovarian cancer, Tfr cells are preferentially enriched within TLS, and an elevated intratumoral Tfr/Tfh ratio is an independent predictor of TLS pre-GC arrest and poor clinical outcome, directly explaining the functional heterogeneity of structurally similar TLS.

#### 4.1.3 Tfc–B cell interactions

Tfc have recently emerged as a subset capable of accessing B-cell follicles and TLS, raising the possibility that they serve as a functional bridge between humoral and cytotoxic immunity. However, current evidence for Tfc–B cell interactions is largely derived from other tumor types, particularly colorectal cancer, and their role in ovarian cancer remains poorly defined. In microsatellite-stable colorectal cancer, Tfc cells interact with immune populations through the MIF–CD74–CXCR4/CD44 axis, and computational analyses reveal significant communication between Tfc cells and GC–like B cells, accompanied by enrichment of TLS-associated gene signatures (73). While these findings suggest that Tfc cells may modulate B-cell differentiation and TLS organization, whether such interactions are functionally operative in ovarian cancer—or instead reflect a state of chronic activation or exhaustion—requires direct experimental validation.

### 4.2 Interactions with APCs

The initiation and functional calibration of follicular T-cell programs are critically dependent on APCs. Through regulation of antigen presentation, co-stimulatory signaling, and cytokine availability, APCs determine whether naïve or activated CD4^+^ T cells are directed toward follicular differentiation or diverted into alternative effector lineages. Although mechanistic studies specifically addressing APC–follicular T-cell interactions in ovarian cancer remain limited, the defining features of this tumor type—including chronic antigen exposure, dysfunctional antigen presentation, and a strongly immunosuppressive cytokine milieu—strongly suggest that this axis represents a major bottleneck constraining effective follicular immunity.

DCs play a dominant role during the early stages of follicular T-cell differentiation. By presenting antigen–MHC class II complexes and providing ICOSL-mediated co-stimulation, DCs promote the differentiation of naïve CD4^+^ T cells into Tfh precursors, whereas subsequent maturation and long-term maintenance of the Tfh program depend on sustained interactions with B cells (61). Genetic or functional disruption of DC-mediated signaling profoundly impairs follicular immunity (74). At the cytokine level, DC-derived signals exert fine control over follicular fate decisions. In human systems, IL-12 and TGF-β synergistically induce Tfh-associated transcriptional programs, while concurrent suppression of IL-2 signaling releases constraints on CXCL13 expression, thereby facilitating follicular chemokine gradients and spatial immune organization (75, 76). These findings highlight the fine balance between differentiation-promoting and inhibitory signals delivered by DCs. Inflammatory cues further modulate DC-driven follicular differentiation. In antiviral models, IFN-γ signaling reprograms DC function and indirectly suppresses Tfh differentiation; blockade of IFN-γ signaling increases Tfh abundance, enhances GC B-cell formation, and augments antibody production (67). These findings suggest that inflammatory cytokines do not act directly on Tfh cells alone but reshape follicular immunity by altering APCs functional states. In ovarian cancer, where persistent IFN-γ exposure and DC dysfunction are common, such mechanisms may actively limit the establishment of robust follicular T-cell programs at early differentiation stages.

Beyond DCs, TAMs represent another important APCs population influencing follicular immunity. M2-polarized macrophages may indirectly shape follicular T-cell differentiation and function through cytokine and metabolic signaling. In models of pulmonary arterial hypertension, increased Tfh cell frequencies are accompanied by M2 macrophage accumulation, whereas suppression of Tfh differentiation reduces both M2 polarization and IL-21 production. Mechanistically, extracellular matrix protein 1 antagonizes IL-2–STAT5 signaling while promoting BCL6 expression, thereby facilitating Tfh differentiation and IL-21 secretion (77). Similarly, in systemic lupus erythematosus models, defective macrophage phagocytosis alters Tfh/Tfr subset balance. While these findings are derived from autoimmune contexts, they suggest that macrophage dysfunction may similarly disrupt follicular immunity in ovarian cancer (78). These observations indicate that macrophage dysfunction can reshape follicular T-cell subset composition and immune output. Given the enrichment of M2-polarized TAMs, impaired phagocytosis, and chronic immunosuppressive inflammation in ovarian cancer, APCs likely not only initiate follicular T-cell differentiation but also continuously modulate their functional state during tumor progression. Through these mechanisms, APCs may influence GC dynamics, TLS maturation, and the overall contribution of humoral immunity to antitumor responses.

### 4.3 Interactions with CD8^+^ T cells

Importantly, through IL-21 production, Tfh cells not only sustain humoral immunity but also potentiate CD8^+^ T-cell effector function, thereby linking antibody-mediated and cytotoxic immune responses. Follicular T cells, particularly Tfh cells, contribute to antitumor immunity by enhancing CD8^+^ T-cell expansion, effector differentiation, and functional persistence. This cooperation is primarily mediated through cytokines such as IL-21 and establishes a functional bridge between humoral and cytotoxic immune responses. In ovarian cancer, however, this positive regulatory axis is frequently attenuated by chronic antigen stimulation, Tfr-mediated suppression, and the immunosuppressive TME (45, 79). As a result, insufficient Tfh support or relative enrichment of Tfr cells may jointly constrain CD8^+^ T-cell effector potential.

Within tumors, follicular T cells and CD8^+^ T cells form a bidirectional regulatory network whose outcome depends on follicular T-cell subset composition, spatial localization, and functional state. Tfh cells promote CD8^+^ T-cell effector differentiation through IL-21 signaling, as IL-21 receptor expression is broadly maintained on tumor-infiltrating CD8^+^ T cells. IL-21 enhances granzyme B and IFN-γ production and supports the maintenance of PD-1^hi effector populations. In tumor models, genetic deficiency of Tfh cells or impairment of IL-21R signaling in CD8^+^ T cells markedly reduce effector CD8^+^ T-cell numbers and accelerates tumor progression. Conversely, adoptive transfer of tumor-specific Tfh cells restores CD8^+^ T-cell functionality and improves tumor control (61). At the transcriptional level, IL-21 regulates exhaustion-associated programs by modulating factors such as T-bet, IRF4, and BLIMP-1. Blockade of IL-21 signaling diminishes Tfh-mediated antitumor effects, reduces IFN-γ, TNF-α, and granzyme B production by CD8^+^ T cells, and reshapes the exhausted T-cell compartment (80). Interestingly, the dependence of exhausted CD8^+^ T-cell subsets on Tfh-derived IL-21 appears to be context dependent. In lymphocytic choriomeningitis virus infection models, Tfh cells contribute to the maintenance of CX3CR1^+^ exhausted-like CD8^+^ T cells; however, partial preservation of this population in the absence of Tfh cells suggests the existence of compensatory IL-21 sources (63). These findings imply that, within tumors, Tfh cells may be particularly important for sustaining functionally competent exhausted-like CD8^+^ T-cell subsets rather than driving terminal exhaustion.

In contrast, Tfr cells exert potent suppressive effects on CD8^+^ T-cell responses. Expression of TGFB1 and IL10 in Tfr cells negatively correlates with IFNG expression in tumor-infiltrating CD8^+^ T cells. *In vitro* co-culture studies using ovarian cancer patient–derived cells demonstrate that Tfr cells suppress CD8^+^ T-cell activation in an IL-10–dependent manner, potentially in synergy with TGF-β signaling (45). In glioma models, Tfr cells exhibit stronger suppressive capacity than conventional Treg cells, particularly against CXCR5^+^ CD8^+^ T cells. If confirmed in ovarian cancer, this would suggest that Tfr-mediated suppression may selectively target the most potent cytotoxic populations within the TME (81). These observations suggest that Tfr-mediated suppression may selectively dampen the most effective cytotoxic populations within the TME. CD8^+^ T cells also actively shape the follicular immune landscape. In several tumor types, including lung cancer, melanoma, colorectal cancer, and breast cancer, CD8^+^ T cells represent a major source of CXCL13, whose expression can be induced by tumor-derived TGF-β. Disruption of the CXCL13–CXCR5 axis—through CXCL13 blockade, CD8^+^ T-cell depletion, or inhibition of TGF-β signaling—reduces intratumoral Tfh accumulation and accelerates tumor growth (80). These findings indicate that CD8^+^ T cells contribute to the establishment of Tfh-enriched immune niches, thereby reinforcing follicular immune circuits. Chronic antigen stimulation further links follicular T cells and CD8^+^ T cells at the clonal and transcriptional levels. In non–small cell lung cancer, Tfh cells exhibit exhaustion-like transcriptional profiles and share clonal relationships with tumor-specific exhausted CD8^+^ T cells and TCF7^+^SELL^+^ progenitors in tumor-draining lymph nodes, characterized by expression of PDCD1, ENTPD1, TNFRSF18, and TNFRSF4 (82). In infection models, IFN-γ derived from CD8^+^ T cells exert only limited inhibitory effects on Tfh differentiation, indicating that follicular T-cell programs are regulated predominantly through IFN-γ–independent mechanisms (67).

Collectively, follicular T cells and CD8^+^ T cells engage in both IL-21–centered cooperative axes and Tfr-mediated suppressive pathways. The balance between these opposing forces may determine whether intratumoral CD8^+^ T cells sustain effective antitumor activity. In ovarian cancer, insufficient Tfh support or relative Tfr enrichment likely constrains CD8^+^ T-cell effector potential.

From a translational perspective, the balance between Tfh-derived IL-21 support and Tfr-mediated suppression may dictate whether tumor-infiltrating CD8^+^ T cells maintain effector competence or progress toward terminal exhaustion, providing a mechanistic basis for patient stratification in immunotherapy.

### 4.4 Interactions with other immune cell types

Follicular T-cell differentiation occurs in competition with alternative CD4^+^ T-cell lineages, particularly Th1 and Th17 cells, with antigen presentation context and B-cell involvement serving as critical determinants of lineage choice. In the presence of cognate B cells, tumor-specific CD4^+^ T cells preferentially adopt a Tfh fate, whereas Th1 differentiation is suppressed; in contrast, B-cell–deficient tumors favor Th1 polarization (61). These findings underscore the central role of B cells in directing follicular immune programs. Within tumors, CD4^+^ T-cell subsets also display substantial clonal overlap and phenotypic plasticity. Single-cell analyses reveal extensive TCR sharing among Th1, Th17, and Tfh populations, with trajectory inference indicating frequent clonal transitions from Th17 to CXCL13^+^ Tfh states. Notably, this clonal continuity is markedly reduced in tumors with restricted differentiation capacity (83), providing a potential explanation for diminished or dysfunctional Tfh compartments in ovarian cancer.

Tfr cells are closely related to conventional Treg in both clonal origin and differentiation trajectory. Single-cell profiling demonstrates extensive TCR sharing between Tfr and Treg populations, with Tfr cells often exhibiting greater clonal expansion. In melanoma, trajectory analyses suggest that activated Treg subsets—particularly those expressing 4-1BB—can differentiate into Tfr cells in response to tumor antigen stimulation (84). Functionally, Tfr cells suppress CD8^+^ T-cell proliferation and cytokine production more potently than conventional Treg cells, highlighting their distinct role in follicular immune regulation.

**Figure 3:**
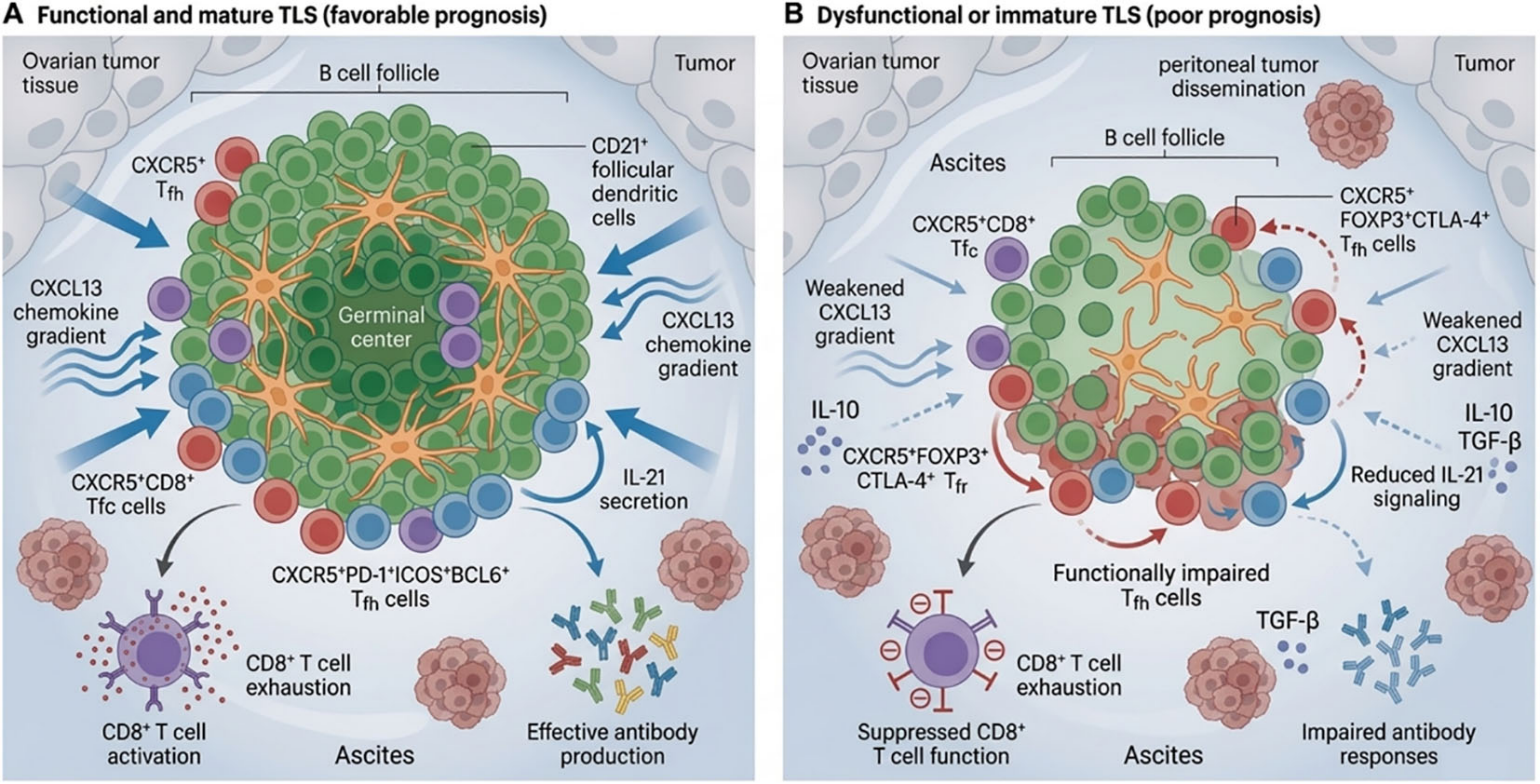
Spatial organization and functional states of tertiary lymphoid structures in ovarian cancer. Tertiary lymphoid structures (TLS) serve as spatially organized immune niches in ovarian cancer, but their functional impact is strongly influenced by the unique tumor microenvironment, particularly the presence of ascites and peritoneal dissemination. **(A)** Functional and mature TLS associated with favorable prognosis. Well-organized TLS exhibit clearly defined B cell follicles supported by a dense CD21^+^ follicular dendritic cell network. CXCR5^+^PD-1^+^ICOS^+^BCL6^+^ Tfh cells localize at the T–B cell interface and within germinal centers, where they provide IL-21–dependent help to B cells, promoting affinity maturation and effective antibody production. CXCL13 gradients facilitate the recruitment of CXCR5^+^ immune cells, including CXCR5^+^CD8^+^ Tfc cells, supporting coordinated follicular immune responses. A balanced presence of CXCR5^+^FOXP3^+^CTLA-4^+^ Tfr cells maintains immune regulation without compromising antitumor activity. **(B)** Immature or dysfunctional TLS in ovarian cancer (commonly observed in HGSOC). In contrast, TLS in ovarian cancer frequently display impaired germinal center formation, reduced structural organization, and an increased Tfr/Tfh ratio. Within the ascites-associated microenvironment, enriched immunosuppressive cytokines such as IL-10 and TGF-β contribute to Tfh dysfunction, limit B cell activation, and restrict CXCR5^+^CD8^+^ T cell infiltration. These alterations result in impaired humoral responses and suppressed cytotoxic immunity. This comparison highlights that TLS dysfunction in ovarian cancer is closely linked to its distinctive microenvironmental conditions rather than merely reflecting structural variability.

These findings suggest that, in ovarian cancer, the functional state of the follicular T-cell network—rather than its mere presence—may be of greater biological and prognostic relevance. Collectively, follicular T cells orchestrate tumor-local immune regulation through multidirectional interactions with B cells, APCs, and cytotoxic lymphocytes (**Figure 3**). The ultimate immunological outcome is governed not by any single cell population but by the dynamic equilibrium between immunostimulatory Tfh cells and immunosuppressive Tfr cells.

## 5 Translational framework: targeting the follicular T cell axis

As our understanding of the ovarian cancer immune microenvironment deepens, it has become increasingly clear that therapeutic strategies focused solely on enhancing effector CD8^+^ T-cell function is insufficient to overcome the limited clinical responsiveness to immunotherapy. Accumulating evidence indicates that the follicular T-cell axis—comprising Tfh, Tfr, and Tfc cells—serves as a central regulatory hub coordinating TLS maturation and integrating humoral and cellular antitumor immunity.

From a translational perspective, therapeutic targeting of the follicular T-cell axis is not intended to simply amplify or suppress a single immune subset. Rather, the goal is to restore coordinated antitumor immunity by reshaping subset composition, functional state, and spatial organization of follicular immune networks. Building on the mechanistic insights outlined above, this section systematically summarizes emerging translational intervention strategies targeting follicular T-cell biology, with the aim of providing a conceptual framework for optimizing immunotherapeutic approaches and guiding future clinical trial design in ovarian cancer (**Table 2**).

**Table 2:** Therapeutic strategies targeting follicular T cell immunity.

Therapeutic level	Specific strategy	Primary targets/pathways	Effects on Tfh cells	Effects on Tfr cells	Implications for antitumor immunity	References
Developmental regulation	BCL6-dependent Tfh differentiation	BCL6, TCF-1	Essential for Tfh maintenance and GC support	Indirect	Promotes TLS formation and limits tumor growth	(63, 64)
	SATB1 modulation	SATB1, ICOS	SATB1 downregulation enhances Tfh differentiation and IL-21/CXCL13 production	SATB1 loss impairs Tfr differentiation	Rebalances follicular helper vs regulatory programs	(47)
	Adoptive Tfh transfer	Ex vivo–expanded Tfh cells	Direct induction of functional Tfh networks	Bypasses endogenous Tfr control	Induces TLS formation and tumor suppression	(66)
Immune checkpoint blockade	PD-1 blockade	PD-1	Preferential activation of PD-1^hi^ Tfh and IL-4 production	Paradoxical expansion of Tfr	Dual effects on antitumor immunity	(80, 84, 87)
	CTLA-4–mediated Tfr depletion	CTLA-4	Indirectly enhances Tfh-driven responses	Reduces Tfr-mediated suppression	Amplifies PD-1 therapeutic efficacy	(78)
	Combined PD-1/CTLA-4 therapy	PD-1, CTLA-4	Induces activated intratumoral Tfh	Alters Tfh expansion dynamics	Shapes synergistic immune responses	(88, 89)
Chemokine axis modulation	CXCL13–CXCR5 axis activation	CXCL13, CXCR5	Enhances Tfh recruitment and follicular positioning	Minimal direct effect	Promotes TLS maturation and CD8^+^ T cell infiltration	(53, 91)
Cytokine and costimulatory pathways	BAFF signaling	BAFF–BAFFR (BR3)	Sustains Tfh differentiation via NF-κB–BCL6	Indirect	Maintains established follicular networks	(93)
	Engineered cytokines	PD1–IL2v	Induces CXCL13 and Tfh/Th1-like programs	Relative restraint	Selectively amplifies functional follicular immunity	(95)
Suppressive pathway targeting	FOXP3 inhibition	FOXP3 (ASO)	Releases helper potential indirectly	Destabilizes Tfr identity	Enhances effector T cell infiltration	(2)
	Exosomal PD-L1 blockade	sEV-PD-L1	Restores Tfh proliferation and IL-21 production	Limits Tfr expansion	Reverses follicular immune suppression	(96)
	VISTA blockade	VISTA	Indirect	Reduces potent Tfr-mediated suppression	Restores CD8^+^ T cell function	(79)
	TNFR2 signaling inhibition	TNFR2	Indirect	Limits Tfr proliferation and stability	Weakens follicular suppressive network	(97)
Metabolic regulation	SIRT3 deficiency/OXPHOS inhibition	SIRT3, OXPHOS	Strongly enhances Tfh differentiation	Context-dependent	Links metabolic state to follicular immunity	(85)
	AMPK activation	AMPK (AICAR)	Restores and amplifies Tfh differentiation	Minimal	Enhances GC reactions and humoral support	(86)
Engineered and cellular therapies	CAR-iTfh cells	BCL6-engineered T cells	Programmable helper function	Controlled	Synergizes with CAR-CD8 therapies	(99)
	Myeloid suppression targeting	PI3Kγ inhibition +α-Enolase vaccine	Indirect	Releases Tfh activation	Indirect	(100)

Tfh, T follicular helper cell; Tfr, T follicular regulatory cell; TLS, tertiary lymphoid structure; GC, germinal center. Effects summarized reflect predominant findings from preclinical models and emerging translational evidence.

### 5.1 Developmental regulation

The differentiation and functional maintenance of follicular T cells are governed by finely tuned transcriptional programs, with BCL6 and FOXP3 acting as the master regulators of Tfh and Tfr cells, respectively. Within the TME, these transcriptional axes collectively shape the direction and quality of follicular immune responses. Although terminal Tfh differentiation requires B-cell interactions, early progenitor populations can be maintained in the absence of B cells, indicating a staged differentiation process dominated by BCL6 and subsequently reinforced by B-cell engagement (63). In CD4^+^Cre Bcl6^fl/fl lung adenocarcinoma models, loss of Tfh cells markedly accelerates tumor growth and disrupts TLS formation (64), providing mechanistic insight into the scarcity of Tfh cells and immaturity of TLS observed in ovarian cancer. In this context, special AT-rich sequence-binding protein 1 (SATB1) emerges as an important upstream regulator of both Tfh and Tfr differentiation. SATB1 restricts Tfh development by directly repressing ICOS expression, whereas TGF-β–mediated downregulation of SATB1 promotes the differentiation of CD4^+^ T cells toward a CXCR5^+^PD-1^hiICOS^+^BCL6^+^ Tfh phenotype and enhances secretion of IL-21, CXCL13, and LIGHT. Concurrently, SATB1 participates in Tfr differentiation through regulation of the Foxp3 enhancer region, and its loss suppresses Tfr development (47).

Collectively, BCL6- and FOXP3-dependent developmental programs not only determine the abundance and functional state of Tfh and Tfr cells but also exert broad effects on TLS organization, humoral immunity, and CD8^+^ T-cell effector function. In ovarian cancer, precise modulation of this developmental axis may therefore offer greater therapeutic leverage than indiscriminate enhancement or depletion of individual immune subsets, providing a mechanistic foundation for targeting follicular T cells as immunotherapeutic nodes.

Functionally, adoptive transfer of BCL6-overexpressing Tfh cells is sufficient to induce *de novo* formation of mature GC-type TLS in immunologically “cold” tumors, an effect strictly dependent on Tfh-derived CXCL13 and IL-21, confirming that Tfh cells are both necessary and sufficient for functional antitumor TLS development. Beyond transcriptional regulation, metabolic reprogramming shapes Tfh fate. The mitochondrial sensor SIRT3 negatively regulates Tfh differentiation via the mTOR-HIF-1α-BCL6 axis (85), while AMPK activation restores Tfh function impaired by chemotherapy (86). These findings suggest that metabolic modulation may rebalance follicular immunity in ovarian cancer.

### 5.2 Immune checkpoint regulation

ICIs exert profound effects on the TME that extend beyond effector CD8^+^ T cells and critically involve the follicular T-cell axis, particularly the balance between Tfh and Tfr cells. Because both subsets express high levels of PD-1 and CTLA-4, they represent direct targets of checkpoint blockade and play a decisive role in shaping the direction and magnitude of post-treatment immune responses. Notably, PD-1 blockade reveals a counter-regulatory effect through Tfr cells. Anti-PD-1 treatment significantly increases intratumoral Tfr frequencies, whereas Tfr-deficient mice exhibit enhanced antitumor responses. Pre-emptive Tfr depletion using anti-CTLA-4 antibodies further amplifies PD-1 efficacy. These findings suggest that PD-1 signaling physiologically restrains Tfr expansion, and its blockade inadvertently enhances Tfr-mediated immunosuppression, partially offsetting CD8^+^ T-cell-driven antitumor effects (84). This mechanism explains the limited efficacy of PD-1/PD-L1 blockade in ovarian cancer.

In contrast, Tfh cells act as key positive regulators of ICI efficacy. In Tfh-deficient mice, antitumor responses to anti-PD-L1 therapy—either alone or combined with adoptive CD8^+^ T-cell transfer—are markedly attenuated, and checkpoint blockade fails to induce organized intratumoral T-cell clustering. These findings indicate that Tfh cells not only provide cytokine support to CD8^+^ T cells but also serve as structural organizers of immune cell architecture, suggesting that Tfh cells may be a critical determinant of checkpoint therapy efficacy in multiple tumor types (80). In T-cell lymphoma and colorectal cancer models, anti-PD-1 therapy preferentially activates PD-1^hi Tfh cells and markedly enhances IL-4 production. Blockade of IL-4 or inhibition of the Tfh master regulator BCL6 abolishes checkpoint-induced immune activation in tumor-draining lymph nodes, whereas exogenous IL-4 is sufficient to recapitulate these effects, identifying the Tfh–IL-4 axis as a critical amplifier of CD8^+^ T-cell responses (87). These observations suggest that impaired Tfh function in ovarian cancer may directly weaken this amplification loop.

Combination immunotherapy further highlights the regulatory role of follicular T cells in therapeutic synergy. In brain metastasis models, combined CTLA-4 and PD-1 blockade induces intratumoral accumulation of highly activated, CTLA-4^hi Tfh cells, whereas Tfh deficiency significantly diminishes the synergistic antitumor effect of dual checkpoint inhibition (88). Analysis of triple-negative breast cancer samples reveals that, compared with PD-1 monotherapy, combination therapy is associated with a smaller increase in intratumoral Tfh proportions, suggesting that combination regimens may reshape the immune microenvironment by altering Tfh expansion or trafficking dynamics (89). Importantly, baseline Tfh status strongly correlates with clinical response: responders exhibit higher frequencies of Ki-67^+^PD-1^+^ Tfh cells in both peripheral blood and tumor tissue prior to treatment, and Tfh abundance correlates positively with tumor regression (89). Similarly, tumor-specific Tfh cells expand in tumor-draining lymph nodes following ICI therapy in colorectal cancer (90). ICIs also integrate follicular immune networks through the CXCL13–CXCR5 axis. In melanoma, anti-PD-1 therapy enhances CXCL13 production, promotes recruitment of CXCR5^+^ B cells, increases B-cell receptor diversity, and coordinately activates Tfh and tumor-reactive CD8^+^ T cells, correlating with prolonged survival (91). In bladder cancer, CXCR5^+^CD8^+^ T cells—characterized by high PD-1 and LAG-3 expression—recover functional activity following PD-1/TIM-3 blockade (92), further linking follicular immune circuits to checkpoint regulation.

Collectively, ICIs influence not only effector CD8^+^ T cells but also follicular T-cell subsets in ways that may profoundly shape therapeutic outcomes in ovarian cancer. Given the high expression of PD-1 and CTLA-4 on both Tfh and Tfr cells, differential subset-specific responses to checkpoint blockade may determine TLS functionality and the overall direction of antitumor immunity. Incorporating follicular T-cell status into response assessment frameworks and exploring rational combination strategies based on follicular immune features may therefore help overcome the limited responsiveness of ovarian cancer to ICI therapy.

Accumulating clinical evidence in ovarian cancer has linked the follicular T cell axis directly to immunotherapy responsiveness. High levels of Tfh infiltration, a favorable Tfr/Tfh ratio, and the presence of functionally mature TLS are associated with higher response rates and prolonged survival in patients treated with ICIs. In contrast, elevated Tfr abundance, Tfh dysfunction, and immature TLS correlate with poor ICI efficacy, reinforcing the notion that the follicular immune status shapes the therapeutic outcome. The CXCL13−CXCR5 axis, which mediates the recruitment and positioning of follicular T cells, further serves as a key indicator of an immune−active tumor microenvironment and predicts improved benefit from PD−1/PD−L1 blockade. Together, these clinical correlations highlight the follicular T cell axis as a promising biomarker for patient stratification and a critical target to overcome immunotherapy resistance in ovarian cancer.

### 5.3 Co-stimulation and cytokine pathways

In ovarian cancer patients, follicular T cells undergo substantial quantitative and functional alterations. Compared with non-tumor controls, peripheral blood CD4^+^CXCR5^+^ Tfh cells from ovarian cancer patients exhibit a significantly increased proportion of PD-1^+^ cells, characterized by enhanced proliferative capacity and elevated secretion of IL-21 and IL-10. PD-1^+^ Tfh cells derived from ovarian cancer patients display markedly higher cytokine-producing capacity than those from non-cancer controls, suggesting that the tumor-associated immune milieu drives polarization toward a functionally active Tfh state. Based on differential expression of PD-1 and TIM-3, Tfh cells can be subdivided into functionally distinct subsets, with PD-1 marking robust helper activity and TIM-3 expression being associated with functional impairment (46), providing a molecular basis for selective Tfh modulation. Insights from autoimmune disease models further inform this axis. B-cell activating factor (BAFF) can directly engage B-cell activating factor receptor (BAFFR, BR3) on T cells, activate NF-κB signaling, and upregulate BCL6 and CXCR5, thereby promoting Tfh differentiation; however, full GC formation requires BAFF signaling in both T and B cells. These observations suggest that persistent BAFF signaling in ovarian cancer may sustain pre-existing Tfh networks and may also explain the limited efficacy of BAFF antagonists in reversing established follicular responses (93).

Chemokine signaling further links Tfh function to antitumor efficacy. In ovarian cancer mouse models, CXCL13 combined with PD-1 blockade significantly suppresses tumor growth and increases intratumoral infiltration of CXCR5^+^CD8^+^ T cells in a CD8^+^ T-cell–dependent manner (53), indicating that Tfh-associated chemokines can amplify effector antitumor immunity while shaping immune architecture. Similar principles apply across disease contexts: in chronic infection models, CXCR5^+^CD8^+^ T cells exhibit enhanced therapeutic potential under PD-1 blockade (94), while in lung cancer, an engineered cytokine (PD1-IL2v) induces CXCL13 expression in PD-1^+^ T cells and activates Tfh/Th1-like transcriptional programs (95). These findings provide a rationale for selectively amplifying functional follicular immune networks in ovarian cancer.

### 5.4 Inhibitory signaling and immune escape mechanisms

In ovarian cancer—particularly HGSOC—the follicular T-cell network is subject not only to activating signals but also to multilayered inhibitory pathways that are frequently co-opted by tumors to promote immune escape. Single-cell transcriptomic analyses reveal marked enrichment of FOXP3^+^ Tregs within HGSOC peritoneal lesions, with stable FOXP3 expression. Downregulation of FOXP3 using antisense oligonucleotides reprograms Tregs toward effector-like phenotypes, increases infiltration of TBET^+^ effector T cells, and enhances the immunogenic effects of neoadjuvant chemotherapy (NACT) (2), highlighting the Treg/Tfr inhibitory axis as a critical barrier to antitumor immunity in ovarian cancer. Suppressive signals are further amplified by tumor-derived extracellular vesicles. Small extracellular vesicles carrying PD-L1 (sEV-PD-L1) from esophageal cancer cells directly inhibit Tfh proliferation, drive polarization toward CTLA-4^+^IL-10^hi suppressive phenotypes, and expand circulating Tfr cells, resulting in reduced IL-21 and IFN-γ production. Importantly, PD-L1 blockade reverses this Tfh/Tfr imbalance (96). Although derived from esophageal cancer models, these findings are highly relevant to ovarian cancer, where ascites and exosome-rich environments are common. At the molecular level, multiple immune checkpoints reinforce Tfr-mediated suppression. V-domain Ig suppressor of T-cell activation (VISTA^+^) Tfr cells from ovarian cancer patients exhibit potent immunosuppressive activity, inhibiting CD8^+^ T-cell proliferation and effector cytokine production, inducing exhaustion, and skewing CD4^+^ T-cell differentiation away from Th1/Th17 lineages (79). In inflammatory models, tumor necrosis factor receptor 2 (TNFR2) signaling is highly active in Tfr cells; TNFR2 agonism enhances Tfr proliferation, stabilizes Foxp3 expression, and strengthens suppression of Tfh and B cells without impairing CXCL13-guided migration (97).

Importantly, follicular immune suppression is not irreversible. Infection models demonstrate that IFN-γ indirectly suppresses Tfh differentiation, and blockade of IFN-γ signaling restores Tfh potential in Tcf-1^+^ progenitors and rescues GC responses (67). In triple-negative breast cancer, dynamic rebalancing among helper immune subsets—including iNK Tfh cells—can partially compensate for reduced Tfh numbers (98). Together, these findings suggest that immune escape in ovarian cancer reflects not only effector cell exhaustion but also multilayered remodeling of the Tfh/Tfr axis. Targeting pathways such as FOXP3, PD-L1-containing exosomes, VISTA, or TNFR2 may therefore rebalance follicular immune networks and provide new entry points for combinatorial immunotherapy.

### 5.5 Engineered follicular T cells

In the context of cellular engineering, Tfh plasticity provides a foundation for next-generation immunotherapies. Enforced BCL6 expression in peripheral T cells enables *in vitro* generation of induced Tfh (iTfh) cells with canonical helper functions, which can be further engineered into tumor-antigen-specific chimeric antigen receptor (CAR)-iTfh cells. These cells enhance CD8^+^ T-cell recruitment and effector function, and their combination with CAR-CD8^+^ T cells outperform conventional CAR-T strategies in solid tumor models (99), offering a novel approach to reconstruct helper immune axes in “cold” tumors such as ovarian cancer. Indirect modulation of immunosuppressive networks may further liberate Tfh function. Targeting myeloid-derived suppressor cells via PI3Kγ inhibition, combined with α-enolase DNA vaccination, significantly enhances Tfh activation and suppresses tumor growth (100), highlighting myeloid suppression as a critical upstream constraint on follicular immunity.

Despite their promise, these approaches remain largely preclinical, and their safety, durability, and feasibility in ovarian cancer patients—particularly with respect to autoimmune toxicity—require careful evaluation prior to clinical translation.

## 6 Follicular immune signatures as predictive biomarkers

Despite substantial advances in immune checkpoint blockade across multiple solid tumor types, the overall clinical benefit for ovarian cancer patients remains limited, underscoring an urgent need for immune-related biomarkers that enable patient stratification and therapeutic response prediction. In recent years, increasing evidence has linked the presence and functional maturity of TLS to immunotherapy responsiveness in diverse cancers. However, binary classification based solely on TLS presence fails to capture the full immunoregulatory potential of these structures (101). In ovarian cancer, emerging data suggest that molecular and cellular features associated with Tfh cells may provide more refined diagnostic and predictive insights. A systematic analysis of candidate diagnostic biomarkers in ovarian cancer identified PFKP and SCRIB as key molecules, with PFKP expression showing a strong positive correlation with Tfh cells and a negative association with immature B cells, while SCRIB correlated positively with plasma cells, memory B cells, and Tfh cells (102). These findings suggest that Tfh-associated metabolic programs and immune architectural features may reflect a more mature and functionally active humoral immune state in ovarian cancer, thereby providing indirect indicators of immune competence beyond conventional TLS enumeration.

Evidence from neoadjuvant treatment settings further reinforces the predictive relevance of Tfh cells. In HGSOC samples obtained after NACT, the density of CXCR5^+^PD-1^+^FOXP3^-^ CD4^+^ Tfh cells is significantly increased and closely associated with TLS formation and maturation (92, 103). NACT-induced endoplasmic reticulum stress and calreticulin exposure promote dense immune infiltration, including Tfh cells, thereby creating permissive conditions for TLS maturation. In turn, mature TLS are strongly associated with enrichment of TCF1^+^PD-1^+^ CD8^+^ T cells—an immune population considered a critical substrate for sensitivity to ICIs (104). Consistent with this concept, in synthetic HGSOC models with high mutational burden, chemotherapy combined with PD-1 blockade significantly improves survival compared with monotherapy, supporting the notion that the Tfh–TLS axis may serve as a structural determinant of benefit from combination immunotherapy. From a mechanistic perspective, insufficient Tfh abundance and incomplete TLS development in HGSOC may represent key barriers limiting immunotherapy efficacy. It has been proposed that HGSOC is generally characterized by a relatively low neoantigen burden, which may impair Tfh differentiation, hinder GC maturation, and restrict the generation of B cells capable of sustaining ICI-sensitive T-cell phenotypes, thereby constraining the effectiveness of conventional PD-1/PD-L1 blockade (105). This framework provides a rational basis for incorporating Tfh-associated metrics into patient stratification strategies in ovarian cancer. Evidence from other tumor types further supports the predictive value of follicular immune signatures. Across multiple solid malignancies, Tfh or Tfh-like populations consistently correlate with immunotherapy responsiveness. In pancreatic and esophageal cancers, intratumoral Tfc cell frequency is positively associated with patient survival and clinical benefit from PD-1/PD-L1 blockade (56, 106). In renal cell carcinoma and melanoma, high Tfh abundance predicts improved response rates to checkpoint inhibition, whereas low Tfh levels combined with high T-cell stress signatures are associated with poor outcomes (44). In gastric cancer, elevated CXCL13 expression strongly predicts ICI responsiveness and closely correlates with TLS presence (83, 107). Together, these findings provide cross-cancer validation for evaluating the predictive value of the Tfh–TLS axis in ovarian cancer.

Gynecologic malignancies offer particularly relevant supporting evidence. In cervical cancer, expression of the stimulator of interferon genes pathway downstream chemokine CCL5 is strongly correlated with Tfh infiltration, suggesting that innate immune activation states influence Tfh recruitment and maintenance (108). In endometrial cancer cohorts, risk models incorporating Tfh infiltration outperform conventional immune models in predicting prognosis and ICI efficacy (109, 110). These observations provide direct gynecologic oncology benchmarks for developing Tfh-based predictive frameworks in ovarian cancer. Importantly, the predictive significance of Tfh cells is context dependent. Collectively, ovarian tumors characterized by mature TLS, high Tfh infiltration, and a low Tfr/Tfh ratio are more likely to support coordinated humoral and cellular immune responses and thus exhibit greater responsiveness to ICIs or combination immunotherapies. In contrast, tumors with immature TLS or prominent Tfr enrichment may display follicle-like structures with limited immunostimulatory capacity, resulting in suboptimal therapeutic responses.

From a translational standpoint, follicular immune signatures offer favorable detectability and clinical feasibility. Quantitative assessment of follicular immune subsets and their spatial organization using multiplex immunohistochemistry, spatial transcriptomics, or flow cytometry may enable development of clinically actionable biomarkers for predicting immunotherapy efficacy. Taken together, current evidence supports follicular T cells as central immunological nodes linking tumor antigenicity, TLS architecture, effector T-cell functionality, and immunotherapy outcomes. In ovarian cancer, Tfh cells and their associated molecular signatures hold promise as multidimensional biomarkers for predicting immunotherapy sensitivity, combination treatment benefit, although their clinical utility will require validation in large-scale, prospective studies.

## 7 Conclusion and future perspective

This review systematically integrates current evidence regarding the differentiation programs, spatial organization, and functional states of follicular T cells within the ovarian cancer TME. Rather than acting in isolation, these cells form a coordinated follicular immune regulatory axis—centered on Tfh, Tfr, and Tfc cells—that collectively shapes the functional maturation of TLS and the overall trajectory of antitumor immune responses. This framework extends beyond the traditional CD8^+^ T cell–centric paradigm and provides a mechanistic explanation for the limited efficacy of immunotherapy observed in ovarian cancer.

From a translational perspective, the functional status of the follicular immune axis may determine whether TLS serve as sites of effective immune activation or evolve into immunosuppressive niches. A composite “follicular immune signature,” incorporating TLS maturity, the degree of Tfh enrichment, and the Tfr/Tfh ratio, holds promise as a candidate biomarker for predicting responsiveness to ICIs and guiding patient stratification. Moreover, multilayered intervention strategies targeting this axis—including combinatorial immune checkpoint modulation, chemokine axis remodeling, metabolic pathway intervention, and engineered cell-based therapies—represent emerging translational avenues to enhance immunotherapeutic efficacy in ovarian cancer.

Future studies should prioritize systematic validation of the functional heterogeneity of follicular T cell subsets in ovarian cancer–specific contexts and leverage spatial omics and multiparametric immune profiling to elucidate their dynamic regulation across different treatment stages. Incorporating follicular immune signatures into clinical trial design and therapeutic response evaluation may facilitate a shift from empiric immunotherapy application toward mechanism-driven precision intervention. Collectively, targeting the follicular T cell axis represents a promising strategy for optimizing immunotherapy and has the potential to deliver more durable clinical benefit for patients with ovarian cancer.
